# Contact activation of coagulation in newly inserted indwelling catheters

**DOI:** 10.1038/s41598-025-04181-3

**Published:** 2025-06-03

**Authors:** Leila Naddi, Caroline Ulfsdotter Nilsson, Karin Strandberg, Thomas Kander

**Affiliations:** 1 Anaesthesiology and Intensive Care, Department of Clinical Sciences, Lund University, Lund, Sweden; 2 Department of Intensive and Perioperative Care, Skåne University Hospital, 221 85 Lund, Sweden; 3 Department of Clinical Chemistry and Pharmacology, Division of Laboratory Medicine, Coagulation Laboratory, University and Regional Laboratories Region Skåne, Malmö, Sweden

**Keywords:** Blood coagulation, Blood coagulation factors, Pre-analytical phase, Indwelling catheters, Central venous catheters, Thrombosis, Medical research, Thrombosis, Laboratory techniques and procedures

## Abstract

The aim of this this cross-sectional observational study was to investigate coagulation and platelet activation in blood collected from newly inserted catheters. Blood samples were collected from newly inserted central venous, peripheral venous and arterial catheters in adult patients. Sample 1 was collected within seconds after insertion. Sample 2 was collected directly after Sample 1 but after proper flush and discard. A selected set of haemostatic assays were performed and the results for Sample 1 and 2 compared per catheter type. In total 10 patients per catheter type were included between December 2021 and June 2022. For central venous catheters, there was a difference in ROTEM NATEM clotting time, clot formation time, α-angle, prothrombin time international normalised ratio, factor VII and thrombin–antithrombin complex, supporting strongly enhanced activation in Sample 1 compared to Sample 2. Peripheral venous catheters and arterial catheters were less prone to activate coagulation. In conclusion, our results support flush and discard ahead of haemostatic assay blood sampling in newly inserted catheters. Furthermore, the results enhance the understanding of central venous catheter-related thrombosis formation.

## Introduction

International guidelines and reviews recommend the first volume of blood to be discarded prior to collection of blood for haemostatic assays from an indwelling catheter and that certain assays should not be sampled from indwelling catheters at all^1–3^. Despite this, clinical routine in many centres is to sample blood directly from both newly inserted as well as catheters in situ without following these recommendations. Probable reasons for this are to avoid patient harm resulting from unnecessary blood loss leading to hospital-acquired anaemia and need for blood transfusions^4–6^, avoidance of extra venipunctures when an existent access to the patient’s bloodstream is already in place, and lack of knowledge of these recommendations.

When foreign material comes into contact with blood it elicits an immediate response from the host’s coagulation system^7–11^. The magnitude and clinical relevance of this response depends on several factors, including the biocompatibility of the material^11–13^ and is probably more pronounced for a newly inserted catheter compared to an older one. However, international guidelines do not discriminate between newly inserted and older catheters, which could possibly have a significant effect on test results^1,3^.

Furthermore, activation of coagulation and subsequent catheter-bound thrombus formation is a common complication to indwelling catheters, especially central venous catheters, leading to an increase in morbidity and mortality^14–19^. The processes that lead to thrombus formation in some cases and not in others, are still not well described.

The primary aim of the present study was to investigate haemostatic activation, measured with ROTEM^®^ (NATEM assay) clotting time (CT) in blood samples collected within seconds (Sample 1) from a newly inserted central venous catheter (CVC), compared to a second blood sample (Sample 2) collected from the same catheter following flush and discard of the initial blood. Secondary aims included the same evaluation for peripheral intravenous catheters (PVCs) and arterial catheters (A-lines), as well as comparisons of carefully selected haemostatic blood tests for measurement of activation between Sample 1 and 2 for CVCs, PVCs and A-lines. A comparison between the different catheter types was also defined as a secondary outcome.

The hypothesis was that Sample 1 would present with a hypercoagulable profile compared to Sample 2.

## Material and methods

### Ethics approval and consent to participate

The study was approved by the Regional Ethical Review Authority, Lund, Sweden (protocol number 2022-00265-01). Signed informed consent was obtained from all participants. The study was performed according to good clinical practice guidelines and the statement of ethical principles in the Declaration of Helsinki. The manuscript was prepared according to the STROBE checklist version 4 for cross-sectional studies^20^.

### Setting and participants

The study was conducted at Skåne University Hospital, Lund, Sweden, between 19 December 2021 and 2 June 2022. Patients ≥ 18 years in need of a CVC, PVC and/or A-line were identified by one of the authors and asked to participate with written informed consent. Patients were selected based on logistic availability, which was random and non-consecutive. The only exclusion criteria was ongoing treatment with direct oral anticoagulants or warfarin.

### Primary outcome

ROTEM^®^ (NATEM) CT for blood from CVCs.

### Secondary outcomes

All other haemostatic assays, described below, from all three catheter types and the comparison between the catheter groups.

### Data collection

Baseline data including age, height, weight, diagnosis, ongoing medication, transfusion of blood products and platelet level were obtained manually the electronic health record.

### Blood sampling and handling

As the aim of this study was to investigate the coagulation and platelet activation profile in the first blood from a newly inserted catheter, guideline recommendations for a discard tube prior to blood sampling were not followed.

All catheters (CVCs, A-lines and PVCs) were inserted without pre-procedural filling with saline. For all catheter types, Sample 1 was collected within seconds from the catheter insertion. CVCs and PVCs were then manually flushed with 20 ml and 10 ml saline respectively, while A-lines were connected to a heparin-free closed sampling and pressure system (Safedraw^®^, Merit Medical, Jordan UT, USA) and the catheter flushed clean. Five mL blood was discarded before Sample 2 was collected from all catheter types. All blood samples were collected directly into low vacuum pressure Vacuette^®^ CTAD tubes (Greiner Bio-One, Kremsmünster, Austria), designed for haemostatic testing containing sodium citrate, theophylline, adenosine and dipyridamole^21^. The tubes were turned eight times after blood collection and kept at 37° Celsius prior to the ROTEM^®^ analyses which were performed within three hours after blood sampling. After the ROTEM^®^ analyses, the remaining blood underwent centrifugation at 4000G for 15 min, before 600–700 μL of plasma from Sample 1 and 2 from each patient was transferred into cryotubes, which were immediately frozen and stored in – 80 °C pending remaining analyses.

### Laboratory methods

ROTEM^®^ analyses were performed in whole blood using recalcified non-activated thromboelastometry (NATEM) according to the manufacturer’s instructions with the following variables: CT, clot formation time (CFT), α-angle and maximum clot firmness (MCF), and were run until completed (60 min). Most ROTEM^®^ assays include strong intrinsic or extrinsic coagulation activation. A stronger coagulation activation might mask more subtle variations of clotting potential. NATEM only involves recalcification without specific activator. Although haemostasis in the NATEM test will mostly be activated by contact with the test vial, the lack of strong activation might mimic a more physiological speed of the clotting process and could potentially detect the more subtle coagulation activation that would be expected from catheter-initiated coagulation activation^22^. CT measures the time from start of the analysis until initial fibrin formation and is mostly affected by coagulation factors and presence of anticoagulants. CFT and α-angle reflect the rate of further thrombus formation. MCF is a measure of clot strength and is primarily affected by platelet count and fibrinogen concentration^23^.

Routine plasma-based coagulation assays: prothrombin time international normalised ratio (PT-INR) was analysed with the Owren method (Medirox AB, Studsvik, Sweden) (PT-INR) and activated partial thromboplastin time (aPTT) with the Actin FSL reagent (Siemens Healthineers, Marburg, Germany). Both assays were performed on Atellica Coag 360 (Siemens Healthineers).

The Owren method for PT-INR measures the activity of factors II, VII and X of the extrinsic pathway^24^. aPTT measures the activity of the intrinsic pathway after contact activation^25^.

Coagulation factors VII (FVII) and XII (FXII) were analysed with clot-based factor analyses on the Atellica Coag 360 (Siemens Healthineers).

FVII is a vitamin K-dependent coagulation factor. FVII interacts with tissue factor and together they are the main activators of secondary haemostasis in response to vessel injuries with bleeding in vivo^26^. FVII and tissue factor also functions as activators of the classical extrinsic pathway of the coagulation cascade, measured with PT-INR.

Activation of FXII is a key step in initiating the intrinsic pathway of coagulation, commonly measured with aPTT. FXII is activated when in contact with a negatively charged surface^27,28^.

Thrombin-antithrombin complex (TAT) was analysed with manual ELISA assay Enzygnost TAT micro (Siemens Healthineers), inter-assay coefficient of variation (CV%) < 10%. TAT is a marker of activated coagulation, with raised levels during prothrombotic states^29–32^.

P-selectin was also analysed with manual Quantikine ELISA (R&D Systems, Minneapolis, USA), inter-assay CV% < 12%. P-selectin is a soluble activated granular platelet membrane protein linked to platelet activation^33,34^.

### Statistical methods

For each catheter type ten patients were recruited. The same patient was allowed to be included in more than one of the catheter groups, but only once per catheter group. For each catheter two blood samples were collected.

Based on a pilot experiment on CVC-blood (*n* = 5, not included in the present report) the effect size for CT was determined to 1.68. With a two-tailed α-error probability of 0.05 and 95% power for Wilcoxon matched pairs signed-rank test, sample size was calculated to 8 per catheter group (G*power, version 3.1). To allow for dropouts and analyses failure, ten patients were included for each catheter group.

All continuous variables are presented with median and interquartile range (IQR) and numbers with (%). Sample 1 and Sample 2 from all catheter types were compared using the Wilcoxon matched pairs signed-rank test. The delta values of Sample 1–Sample 2 for each variable were compared between the three catheter types using the Kruskal–Wallis test with Dunn’s multiple comparisons test.

In the CVC-group, Sample 1 from two patients showed visible clot formation in the CTAD tube. Due to this, a sensitivity analysis with these samples excluded was performed.

A *P*-value of < 0.05 was considered statistically significant for all analyses.

All statistics were performed with GraphPad Prism version 10.2.2 for Macintosh (GraphPad Software, Boston, Massachusetts USA, http://www.graphpad.com), which was also used for generating all figures.

As there were no missing data there was no need to compensate for this.

## Results

**Fig. 1:**
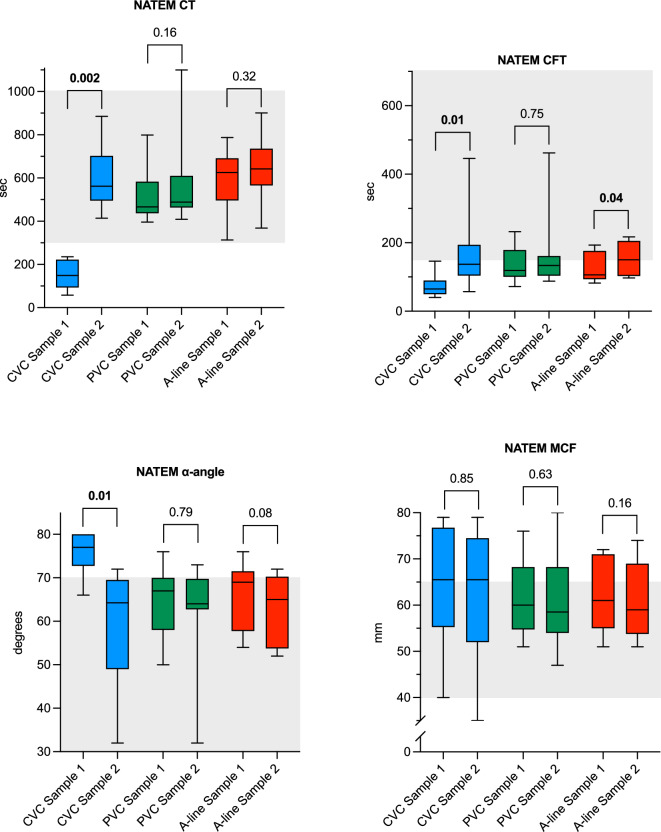
Box plots of whole-blood NATEM assays comparing Sample 1 and Sample 2 for each catheter group. Whiskers represent min/max values. Shaded areas illustrate reference intervals. CT: clotting time; CFT: clot formation time; MCF: maximum clot firmness; CVC: central venous catheter; PVC: peripheral venous catheter; A-line: arterial catheter.

**Fig. 2:**
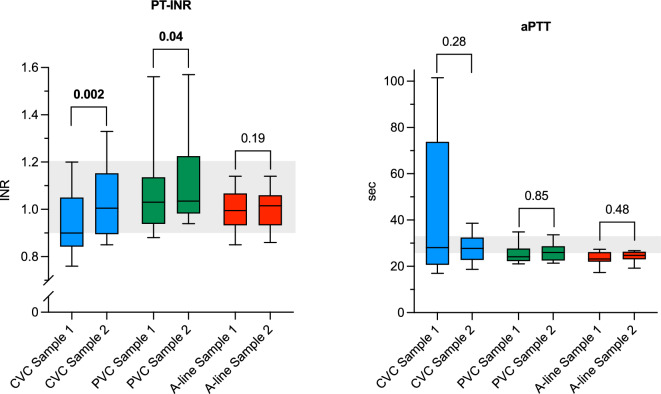
Box plots of routine coagulation assays comparing Sample 1 and Sample 2 for each catheter group. Whiskers represent min/max values. Shaded areas illustrate reference intervals. PT-INR: prothrombin time international normalised ratio; aPTT: activated partial thromboplastin time; CVC: central venous catheter; PVC: peripheral venous catheter; A-line: arterial catheter.

**Fig. 3:**
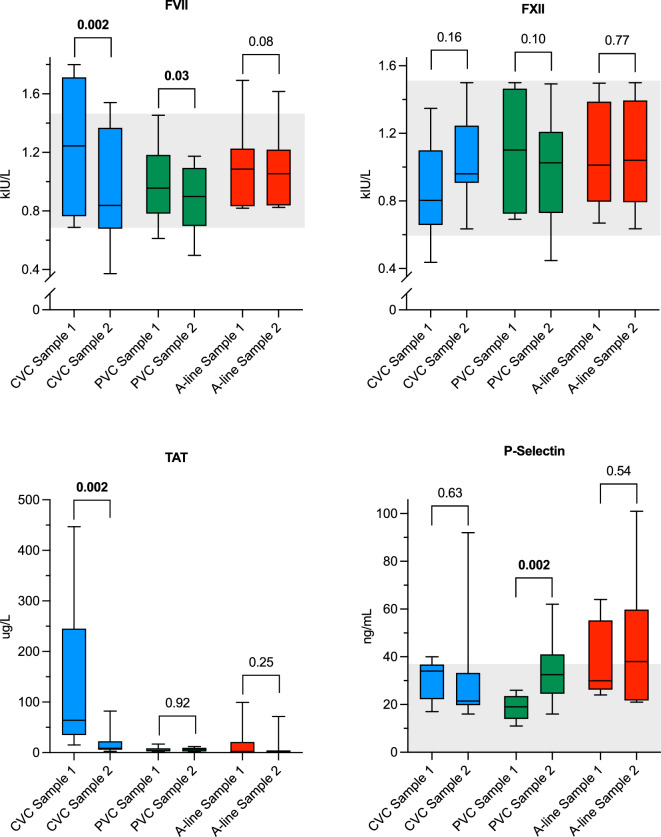
Box plots of special coagulation assays comparing Sample 1 and Sample 2 for each catheter group. Whiskers represent min/max values. Shaded areas illustrate reference intervals. Reference interval for TAT: 1.0–4.1 μg/L. FVII: factor VII; FXII: factor XII; TAT: thrombin-antithrombin complex; CVC: central venous catheter; PVC: peripheral venous catheter; A-line: arterial catheter.

**Fig. 4:**
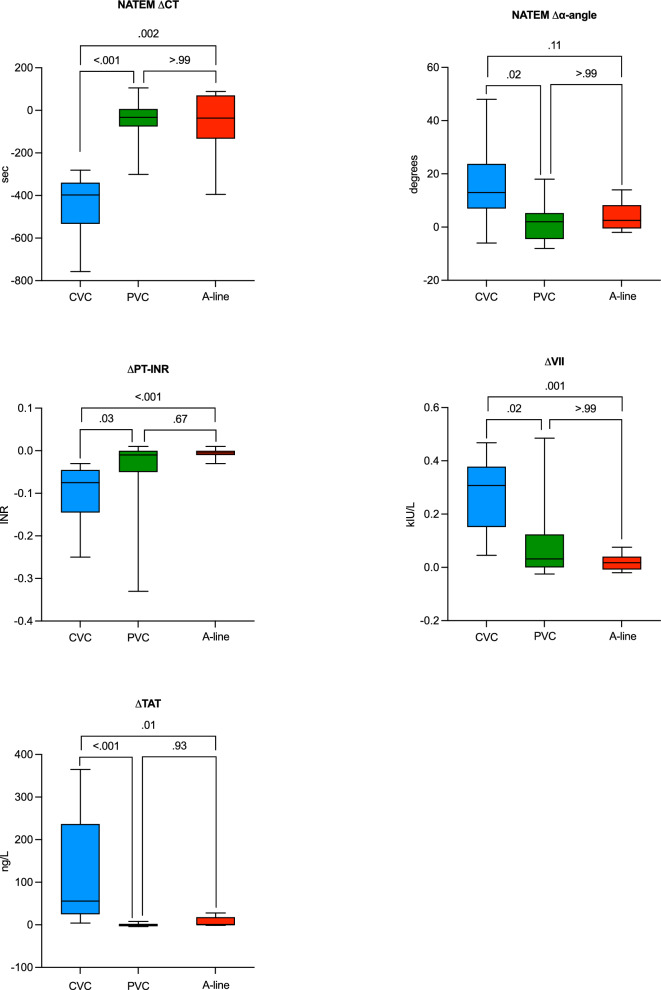
Box plots depicting the Dunn’s multiple comparisons test of the three catheter groups. Whiskers represent min/max values. CT: clotting time; PT-INR: prothrombin time international normalised ratio; FVII: factor VII; TAT: thrombin-antithrombin complex; CVC: central venous catheter; PVC: peripheral venous catheter; A-line: arterial catheter.

A total of 60 blood samples from 24 patients were collected and analysed. Patient and catheter characteristics are reported in Table 1. An overview of the results of all haemostatic assays can be found in Supplementary Table S1. The results from the NATEM analyses are illustrated in Fig. 1, PT-INR and aPTT in Fig. 2, and the other coagulation assays in Fig. 3. Figure 4 illustrates the comparisons between the three catheter types for the variables with demonstrated differences. The remaining comparisons are found in Supplementary Fig. S1.

**Table 1:** Patient and catheter characteristics.

Characteristics	CVC^1^, *n* 10	PVC^2^, *n* 10	A-line^3^, *n* 10
Female sex*, n* (%)	5 (50)	5 (50)	8 (80)
Age (yr), median (IQR)	61 (50–78)	67 (57–67)	63 (54–76)
BMI^4^ (kg/m^2^), median (IQR)	27 (24–30)	27 (23–29)	27 (22–29)
CVC^5^ material, lumen*, n* (%)			
Polyurethane, 1 lm	3 (30)	–	–
Polyurethane^6^, 2 lumens	7 (70)		
CVC length, cm, n (%)			
15	7 (70)		
16	2 (20)		
20	1 (10)		
PVC material, size, *n* (%)			
Polyurethane, 22G		5 (50)	
Polyurethane, 18G	–	3 (30)	–
Polyurethane, 17G		2 (20)	
A-line material, size, *n* (%)			
Polytetrafluoroethylene, 20G	–	–	10 (100)
A-line blood sampling system material, *n* (%)			
Phthalate-free polyvinyl chloride	–	–	10 (100)
Platelet count (× 10^9^/L), median (IQR)	209 (62–265)	261 (218–308)	258 (228–299)
LMWH^7^, prophylactic dose, *n* (%)	2 (20)	6 (60)	7 (70)
LMWH, treatment dose, *n* (%)	1 (10)	0 (0)	0 (0)
ASA/NSAID^8^, ongoing treatment, *n* (%)	1 (10)	2 (20)	1 (10)
Platelet transfusion < 24 h, *n* (%)	1 (10)	0 (0)	0 (0)
Malignant diagnosis, *n* (%)	9 (90)	8 (80)	10 (100)
Solid tumour	4	8	10
Non-solid tumour^9^	5	0	0

^1^Central venous catheter, ^2^peripheral venous catheter, ^3^arterial catheter, ^4^body mass index, ^5^nine CVCs were successfully inserted on the first attempt, one on the second, ^6^including two with outer noble metal coating, ^7^low molecular weight heparin ^8^acetylsalicylic acid/nonsteroidal anti-inflammatory drugs, ^9^leukemia and lymphoma.

Overall, the analyses on CVC-blood demonstrated a hypercoagulable state in Sample 1 compared to Sample 2. The median CT for CVC Sample 1 was 149 (94–222) s, compared to 562 (496–702) s in Sample 2 (*P* = 0.002). Similar results were found for CFT and α-angle, but MCF was not affected. Additionally, all values for CT and CFT in Sample 1 from CVCs were below the lower range limit. Signs of a hypercoagulable state in Sample 1 were also shown for PT-INR, with lower values compared to Sample 2, as well as higher levels of both FVII and TAT in Sample 1 compared to Sample 2. The levels of aPTT, FXII and P-selectin did not differ between the samples.

Blood collected from PVCs did not show any differences for any of the NATEM variables, aPTT, FXII or TAT, but smaller differences were seen in PT-INR and FVII. PVCs were the only catheter type with a demonstrated difference for P-selectin, with markedly higher values in Sample 2 compared to Sample 1.

In the A-line-group only CFT showed a small difference between Sample 1 and Sample 2, while there were no differences for remaining NATEM-assays, PT-INR, aPTT, FVII, FXII, TAT and P-selectin.

The sensitivity analysis, with the two samples with visible clot formation in the CTAD tube excluded, showed essentially the same results as the main analyses (Supplementary Fig. S2).

When comparing the three catheter groups, the CVC group showed a greater difference in activated plasma coagulation between Sample 1 and Sample 2, measured with ΔCT (*P* < 0.001), Δα-angle (*P* = 0.01), ΔPT-INR (*P* < 0.001), ΔFVII (*P* = 0.001) and ΔTAT (*P* < 0.001), compared to both the PVC and A-line groups (Fig. 4). No differences were shown between PVCs and A-lines.

## Discussion and limitations

In this cross-sectional observational study, we have demonstrated that the first blood collected from newly inserted CVCs exhibits a markedly increased plasma coagulation compared with blood collected after flush and discard. The results confirm the hypothesis that Sample 1 would present with a hypercoagulable profile compared to Sample 2 for CVCs but discard the hypothesis for PVCs and A-lines. The ability of CVCs to induce a hypercoagulable state and promote thrombus formation has previously been described^17,35–40^. Our results contribute to a more detailed understanding of the underlying process of the development of CVC-related thrombosis, a well-known complication to CVC-use. Additionally, the results highlight the importance of correct blood sampling techniques to minimize preanalytical errors and ensure accurate results of blood analyses. The results from the comparison between the three catheter groups add to the evidence that CVCs activate coagulation to a much greater extent than the other catheter types.

The demonstrated differences in CT, CFT and α-angle in CVC blood, coupled with the absence of an effect on MCF, suggest an activated coagulation process without significant impact on clot strength. This finding aligns with the understanding that while CT and CFT are influenced by the initiation and propagation phases of coagulation, MCF primarily reflects the contribution of platelet count, fibrinogen concentration, and Factor XIII activity to clot formation and stability. The lack of variation in MCF is therefore expected, given that these factors are unlikely to be substantially altered in the context of our study conditions.

Factor VII activity was increased in Sample 1 compared to Sample 2 for CVCs, but there was no difference in FXII activity. This finding is surprising as FXII is traditionally recognized for triggering coagulation when blood comes into contact with a negatively charged surface^27,28,41^. However, recent literature provides a theoretical basis for the observed results. For example, Konrath et al. demonstrated that surface-activation of FXII can normalise a prolonged aPTT in prekallikrein-deficient plasma, supporting the concept that FXII activity could be increased under such conditions^42^. Given that we measured FXII activity (in kIU/L) and not antigen levels, and considering that antigen levels generally correlate with activity, the observed lack of difference suggests either limited FXII activation or rapid consumption and clearance.

Moreover, coagulation can also be initiated through FVII activating protease when blood comes into contact with a positively charged surface^8,41^. This aligns with our finding of the increased FVII activity, which we interpret as a result of a surface-mediated activation distinct from classical FXII-dependent pathways. These mechanisms can drive coagulation in response to specific surface properties supporting our overall interpretation.

The variability in aPTT values observed in CVC Sample 1 likely reflects inter-individual differences in the extent of coagulation activation immediately following catheter insertion. Factors such as variable degree of coagulation activation by catheter material may contribute to this variability. Notably, Sample 2 values showed much less variation, supporting the notion that the effect is driven by preanalytical artefacts introduced by early blood sampling. This further supports our conclusion that flush and discard are necessary to avoid misleading results in coagulation assays.

Collection of blood from a central or intravenous line is not recommended when measuring prothrombin activation products such as TAT complex^1^. In this study the significant change in TAT levels between Sample 1 and 2 in the CVC group underlines this recommendation.

In the current study two CVCs had an outer noble metal coating, and it was in the Sample 1-blood from these CVCs that visible clots were seen. Although the polyurethane, noble metal coated CVCs are non-coated on the inner surface, contact-activation related to noble metal coating has previously been demonstrated in a study by Stevens et al.^13^. It should also be observed that polyurethane encompasses a broad category of polymers characterized by diverse chemical compositions and properties, and the polyurethane composition on the inside of the noble metal coated CVC may not be the same as the polyurethane in the non-coated CVCs^11,43^.

For PVCs and A-lines only a few assays demonstrated any differences, and none as marked as for CVCs. This may be explained by the larger surface area of the CVC exposed to blood compared to the thinner and shorter PVCs and A-lines^44^. Alternatively, the composition of the plastic material in the included CVCs may be more thrombogenic compared to the plastic materials in the PVCs and the A-lines. This remains to be established, but the results underline the importance of discarding a sufficient volume of blood from indwelling catheters before blood sampling for coagulation analyses.

For A-lines, Sample 2 was collected through the connected heparin-free closed sampling system. Although this provides a greater contact area for the blood before the sample is retrieved, compared to sampling blood directly from the much shorter A-line, only CFT demonstrated a statistically significant difference between Sample 1 and 2. This suggests that the plastic material of the heparin-free closed sampling system in the current study did not activate plasma coagulation.

Although ΔP-selectin did not differ between the catheter-groups, a substantial portion of the A-line samples ended up above the upper reference limit, indicating increased platelet activation in both Sample 1 and 2. It has previously been shown that small-gauge needles tend to increase platelet activation^3^, which could explain the higher levels of P-selectin in A-line samples. In contrast to this, the PVC samples, half of which were drawn from catheters with a smaller lumen than the A-lines, did not demonstrate the same pattern for P-selectin. The reason for the difference in P-selectin between Sample 1 and 2 in blood drawn from PVCs remains unclear.

The vast majority of patients in this study had a malignant diagnosis, a factor that is well-recognized for its potential to contribute to a general hypercoagulable state^45,46^. While this underlying prothrombotic condition does not negate the demonstrated differences in coagulation parameters it could contribute to the magnitude and significance of them and in making the results less generalisable.

This study has several limitations. Given the observational design causal relationships cannot be established. Also, the risk of bias and confounding factors must be considered as this was not a randomised study, with the small sample size adding to the uncertainty of the results. Furthermore, ex-vivo analyses of in-vivo coagulation have limitations as they cannot fully replicate the dynamic physiological conditions present within the circulatory system, including blood flow, shear stress, and the interactions between vascular endothelium and circulating cells. Results from the secondary outcomes should be interpreted with caution, as no sample size calculation was performed for these outcomes. Additionally, there is an increased risk for type 1 error due to multiple testing. Lastly, although sampling and analyses of blood were performed in a standardised manner, small preanalytical variations cannot be ruled out.

## Conclusions

This study showed that coagulation is activated in blood drawn immediately from a newly inserted CVC compared to blood drawn from the same catheter after flush and discard. We found less evidence of haemostatic activation between the two methods of sampling for PVCs and almost none for A-lines. The results add to the knowledge on how CVC-related thrombi arise, as the same activation of coagulation observed in blood exposed to the inside of polyurethane catheters, likely also occurs on the catheter’s exterior surface. Furthermore, the results enhance the practice of discarding an initial volume of blood before sampling for haemostatic assays, particularly when a CVC is used for blood collection.

## Supplementary Information

Supplementary Information.

## Data Availability

The data used and/or analysed during the study are available from the corresponding author upon reasonable request.
