# Supplementary material for: Contact activation of coagulation in newly inserted indwelling catheters
Source: Sci Rep. 2025 Jun 3;15:19378. doi: 10.1038/s41598-025-04181-3 (PMC12134338; doi:10.1038/s41598-025-04181-3)

Title: Contact activation of coagulation in newly inserted indwelling catheters

Leila Naddi[1,2], Caroline Ulfsdotter Nilsson[1,2], Karin Strandberg[3], Thomas Kander[1,2]

[1]Anaesthesiology and Intensive Care, Department of Clinical Sciences, Lund University, Lund, Sweden, [2]Department of Intensive and Perioperative Care, Skåne University Hospital, Lund, Sweden, [3]Department of Clinical Chemistry and Pharmacology, Division of Laboratory Medicine, Coagulation Laboratory, University and Regional Laboratories Region Skåne, Malmö, Sweden.

Supplementary Table S1

Overview of haemostatic assays*

**Haemostatic assay**	**Reference interval**	**CVC[1] Sample 1**	**CVC Sample 2**	***P* value**	**PVC[2] Sample 1**	**PVC Sample 2**	***P* value**	**A-line[3] Sample 1**	**A-line Sample 2**	***P* value**
NATEM CT[4], sec	300-1000	149 (94-222)	562 (496-702)	**.002**	466 (437-583)	489 (463-610)	.16	626 (496-692)	642 (566-736)	.32
NATEM CFT[5], sec	150-700	65 (50-90)	137 (104-194)	**.01**	119 (101-179)	134 (104-161)	.75	107 (94-176)	150 (103-206)	**.04**
NATEM α-angle, °	30-70	77 (73-80)	64 (49-70	**.01**	67 (58-70)	64 (63-70)	.79	69 (58-72)	65 (54-70)	.08
NATEM MCF[6], mm	40-65	66 (55-77)	66 (52-75)	.85	60 (55-68)	59 (54-68)	.63	61 (55-71)	59 (54-69)	.16
PT-INR[7], INR	0.9-1.2	0.9 (0.8-1.1)	1.0 (0.9-1.2)	**.002**	1.0 (0.9-1.1)	1.0 (1.0-1.2	**.04**	1.0 (0.9-1.1)	1.0 (0.9-1.1)	.19
aPTT[8], sec	26-33	28 (21-74)	28 (23-32)	.28	24 (22-28)	26 (23-29)	.85	23 (22-26)	25 (23-26)	.48
Factor VII, kIU/L	0.7-1.5	1.2 (0.8-1.7)	0.8 (0.7-1.4)	**.002**	1.0 (0.8-1.2)	0.9 (0.7-1.1)	**.03**	1.1 (0.8-1.2)	1.1 (0.8-1.2)	.08
Factor XII, kIU/L	0.6-1.5	0.8 (0.7-1.1)	1.0 (0.9-1.2)	.16	1.1 (0.7-1.5)	1.0 (0.7-1.2)	.10	1.0 (0.8-1.4)	1.0 (0.8-1.4)	.77
TAT[9], μg/L	1.0-4.1	64 (35-245)	8.6 (5.9-22)	**.002**	4.1 (2.6-8.7)	6.4 (2.4-9.6)	.92	2.3 (1.0-21)	2.2 (1.0-4.5)	.25
P-Selectin, ng/mL	0-40	34 (22-37)	22 (20-33)	.63	19 (14-24)	33 (25-41)	**.002**	30 (26-55)	38 (22-60)	.54

*Results presented as median (IQR). [1]Central venous catheter, [2]Peripheral venous catheter, [3]Arterial line, [4]Clotting time, [5]Clot formation time, [6]Maximum clot firmness, [7]Prothrombin time international normalised ratio, [8]Activated partial thromboplastin time, [9]Thrombin–antithrombin complex

Supplementary Fig. S1

Box plots depicting the Dunn’s multiple comparisons test of the three catheter groups for clot formation time (CFT), maximum clot firmness (MCF), activated partial thromboplastin time (aPTT), Factor XII (FXII) and P-selectin. Whiskers represent min/max values. Abbreviations: CVC = central venous catheter, PVC = peripheral venous catheter, A-line = arterial catheter.

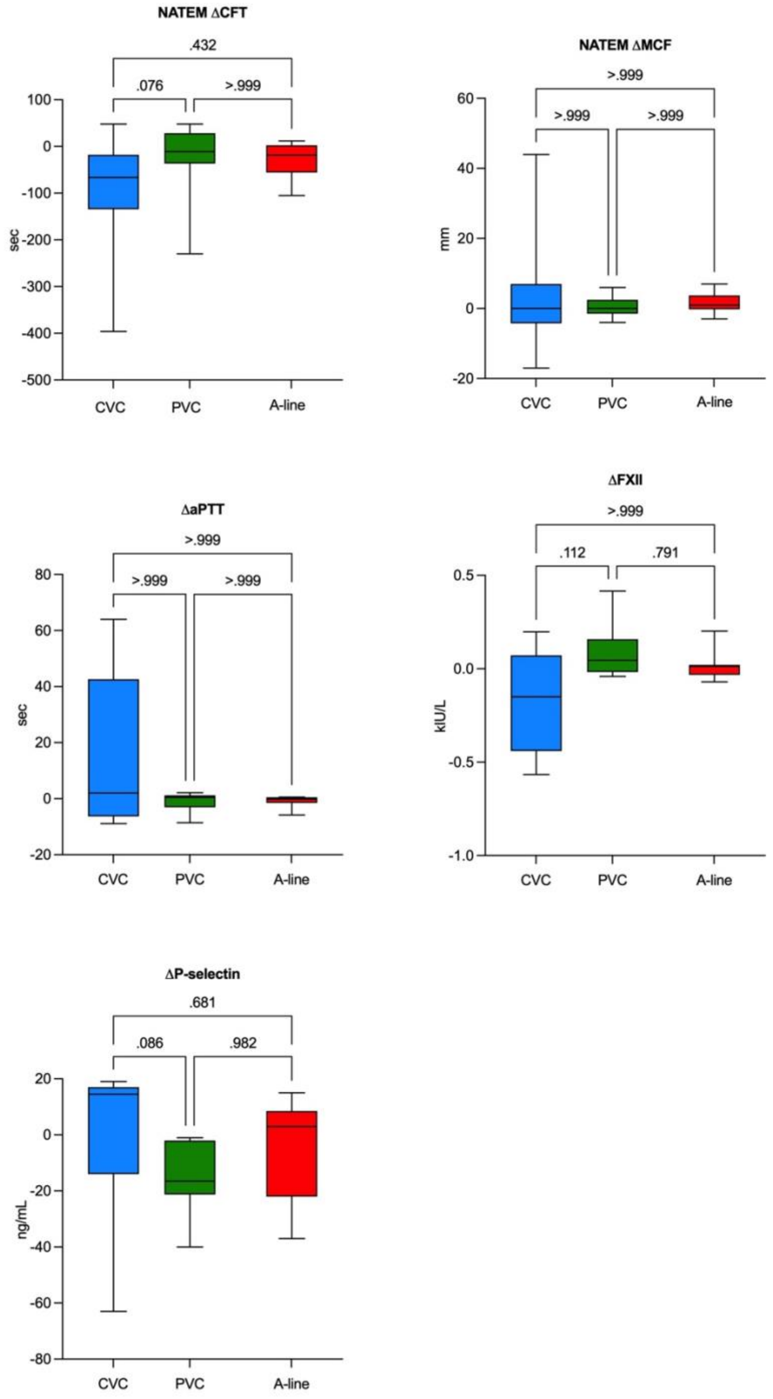

Supplementary Fig. S2

Box plots of the results from the sensitivity analysis of central venous catheter (CVC) samples with two patients excluded due to visible clot formation in Sample 1. Whiskers represent min/max values. Abbreviations: CT = clotting time, CFT = clot formation time, MCF = maximum clot firmness, PT-INR = prothrombin time international normalised ratio, aPTT = activated partial thromboplastin time, FVII = factor VII, FXII = factor XII, TAT = thrombin-antithrombin complex, PVC = peripheral venous catheter, A-line = arterial catheter.

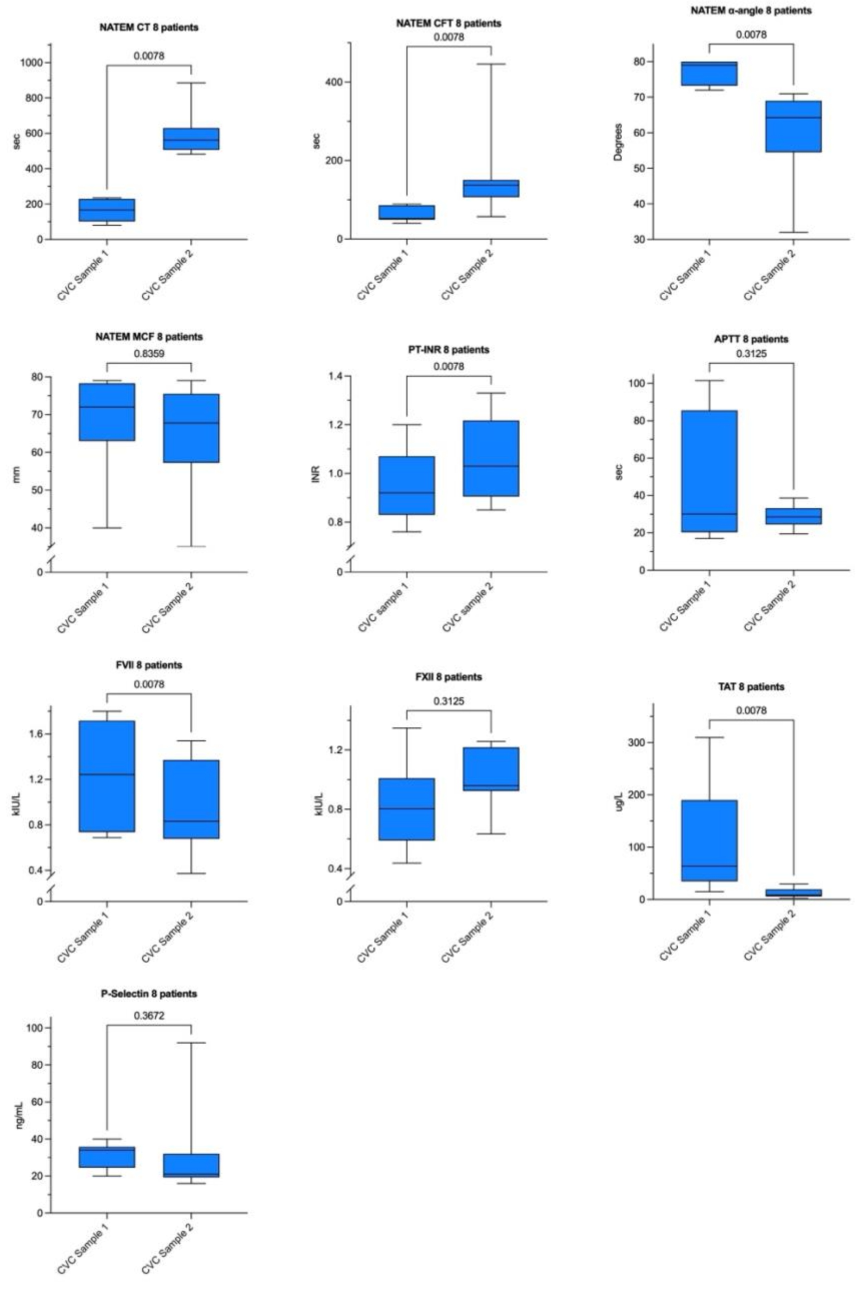

Supplement: Supplementary file 1 — Supplementary Information. [file 41598_2025_4181_MOESM1_ESM.pdf]
